# An Increase in Kinematic Freedom in the Lokomat Is Related to the Ability to Elicit a Physiological Muscle Activity Pattern: A Secondary Data Analysis Investigating Differences Between Guidance Force, Path Control, and FreeD

**DOI:** 10.3389/frobt.2019.00109

**Published:** 2019-10-31

**Authors:** Tabea Aurich-Schuler, Rob Labruyère

**Affiliations:** ^1^Rehabilitation Center for Children and Adolescents, University Children's Hospital Zurich, Affoltern am Albis, Switzerland; ^2^Children's Research Center, University Children's Hospital Zurich, Zurich, Switzerland

**Keywords:** children, patients, robot-assisted gait therapy, surface electromyography, muscle activity, step variability, FreeD, gait

## Abstract

**Background:** Robot-assisted gait therapy is a fast-growing field in pediatric neuro-rehabilitation. Understanding how these constantly developing technologies work is a prerequisite for shaping clinical application. For the Lokomat, two new features are supposed to increase patients' movement variability and should enable a more physiological gait pattern: *Path Control* and *FreeD*. This work provides a secondary data analysis of a previously published study, and looks at surface electromyography (sEMG) during *Guidance Force* walking and six sub-conditions of *Path Control* and *FreeD*.

**Objective:** The aim was to evaluate different levels of kinematic freedom on the gait pattern of pediatric patients by modulating settings of *Path Control* and *FreeD*.

**Methods:** Fifteen patients (mean age 16 ± 2 years) with neurological gait disorders completed the measurements. We analyzed sEMG amplitudes and the correlation of sEMG patterns with normative data of five leg muscles during walking conditions with increasing kinematic freedom in the Lokomat. The new outcome measure of inter-step similarity is introduced as a proxy for walking task complexity.

**Results:** Within *Path Control*, sub-conditions showed significantly higher sEMG amplitudes in a majority of muscles with increasing kinematic freedom, and correlations with the norm pattern increased with increasing kinematic freedom. *FreeD* sub-conditions generally showed low or even negative correlations with the norm pattern and a lower inter-step similarity compared to *Guidance Force*.

**Conclusions:** In general, this work highlights that the new hard- and software approaches of the Lokomat influence muscle activity differently. An increase of kinematic freedom of the walking condition led to an increase in muscular effort (*Path Control*) or to a higher step variability (*FreeD*) which can be interpreted as an increased task complexity of this condition. The inter-step similarity could be a helpful tool for the therapist to estimate the patient's state of strain.

## Introduction

For several years now, robot-assisted therapy devices have established their place in a wide range of interdisciplinary therapies in neuro-rehabilitation. Ever since their introduction, research groups targeted the question, if these robot-assisted therapies might be more effective than conventional therapies. Meanwhile, the opinion has become established that a combined application of both methods is to be recommended (Dundar et al., 2014; Mehrholz et al., 2017).

In pediatrics, however, research is lagging behind. One problem is the lack of guidelines regarding the best application of these devices in children, as well as a paucity of methodologically well-performed randomized controlled trials (Wu et al., 2017). Furthermore, the population pool is small and shows a very heterogeneous clinical appearance. To address this issue, a group of European experts recently published generalizable, practical recommendations (Aurich-Schuler et al., 2015) for the application of the pediatric Lokomat (Hocoma AG, Volketswil, Switzerland), which is one of the most sold robotic therapy devices worldwide. Other recommendations focus on common robotic therapy devices for children and adolescents undergoing neuro-rehabilitation (van Hedel and Aurich-Schuler, 2016). Both publications point out that broad practical know-how is essential, especially for the rehabilitation of young patients. Additionally, it is important to completely understand the mechanisms underlying these technologies (Aurich-Schuler et al., 2015, 2017, 2019; van Hedel and Aurich-Schuler, 2016).

Since it is known that robotic therapy devices also entail disadvantages (e.g., limited variability of movements, many movement restrictions, limited usability for different degrees of impairment, the passivity of patients Riener et al., 2005; Reinkensmeyer et al., 2009; Duschau-Wicke et al., 2010b; Krishnan et al., 2013), technologies to overcome these shortcomings have grown immensely over the last years. Also for the Lokomat, two new soft- and hardware approaches were developed, *Path Control*, and *FreeD*. Both features should increase the patient's participation, allow the patient to walk with more kinematic variability, and a more normal/physiological pattern. *Path Control* offers a spatiotemporal tunnel around the predefined gait trajectory in the sagittal plane which allows a certain degree of individualization (Duschau-Wicke et al., 2010a; Schück et al., 2012). *FreeD* performs an actuated movement of the pelvis of the patient (coupled lateral translation and transverse rotation) and allows mediolateral movements of the leg cuffs (Aurich-Schuler et al., 2017, 2019).

The analyses of the current study base on the data of a research project titled “Can Lokomat therapy with children and adolescents be improved? An adaptive clinical pilot trial comparing *Guidance force, Path control*, and *FreeD*,” parts of which have already been published (Aurich-Schuler et al., 2017). There, an extensive technical background provides insight into the application of these technologies in daily clinical life. The focus of the first paper was on the three control conditions *Guidance Force, Path Control, and FreeD*. The aim was to investigate the differences between these three conditions from the perspective of increasing kinematic freedom on surface electromyography (sEMG) measurements of different leg muscles. Accordingly, settings were chosen in a way that accentuated these differences (*Guidance Force* was very restrictive and *FreeD* was very free). Results showed that with *Path Control*, patients could walk in an active and physiological way (similar to a normal overground walking pattern as compared to a reference curve of healthy children). In contrast, *FreeD* walking resulted in non-physiological muscle activity compared to norm patterns. The settings of the *FreeD* condition seemed to be too difficult (or the task too complex) for the patients. It was further outlined that future studies should investigate the modulations of *FreeD* and the applicability in children and adolescents with neurological gait disorders.

With the present manuscript, we intend to address this by providing data on six sub-conditions with different technical modulations (variation of the support force, freedom for weight shifting, and pelvic rotation/translation). The aim was to evaluate if an increase in kinematic freedom during Lokomat walking within the control conditions *Path Control* and *FreeD*, respectively, leads to enhanced cardiovascular and muscular activity. We were also interested to see if this muscular activity occurs in a physiological way. Additionally, we wanted to assess if the level of task complexity of the walking conditions is related to kinematic freedom with the new outcome measure of inter-step similarity.

## Materials and Methods

### Participants

Sixteen patients (mean age 16 ± 2 years) with neurological gait disorders participated in this clinical pilot study. All participants were experienced Lokomat walkers. Diagnoses were: Cerebral palsy (*n* = 9, 1 × Gross Motor Classification System (GMFCS, Palisano et al., 1997) level I, 3 × GMFCS level II, 4 × GMFCS level III, 1 × GMFCS level IV), acquired brain injury (*n* = 5), meningomyelocele (*n* = 1), hereditary spastic paraplegia (*n* = 1). Since in one participant (CP, GMFCS level IV), the measurements had to be stopped immediately after the beginning due to safety reasons (the patient was not able to walk in the Lokomat with reduced Guidance Force), only data of 15 patients were analyzed. Further characteristics of the patients as well as in- and exclusion criteria can be found elsewhere (Aurich-Schuler et al., 2017).

### Measurements

This study was approved by the Cantonal Ethics Committee of Zurich. All participants ≥14 years and all legal guardians gave written informed consent to participate, participants <14 years provided assent.

**Figure 1:**
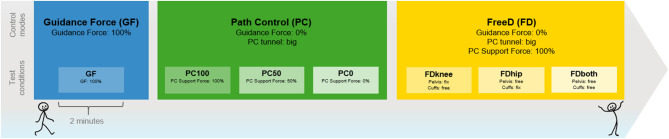
Test procedure. The illustrated order helps to understand the increase of kinematic freedom. During the tests, the order was block-randomized. Each condition lasted 2 min with a 1-min break in between. GF, Guidance Force condition (blue); PC, Path Control condition (green), FD, FreeD condition (yellow).

All measurements were part of our first research project (Aurich-Schuler et al., 2017), and were conducted at the Rehabilitation Center for Children and Adolescents in Affoltern am Albis, Switzerland. Our research group has all the necessary permissions to investigate the trademark registered product Lokomat. The application of the Lokomat and the individual settings for the speed and bodyweight support were also identical (average speed ± standard deviation was 1.96 ± 0.15 km/h; body weight support was 30% of the patient's body weight). A total of seven walking conditions were performed, and each lasted 2 min with 1-min breaks in between. The three major conditions were randomized first, and all sub-conditions within the major conditions were block-randomized thereafter (Figure 1). During *Path Control*, the sub-conditions differed in the amount of support given in following the gait trajectory [support force set to 100% (= PC100), 50% (= PC50), or 0% (= PC0)]. During *FreeD*, the sub-conditions differed regarding the fixation of pelvis and cuffs with according different kinematic freedom (“cuffs free”: the cuffs were set to move mediolaterally (= FDknee); “pelvis free”: actuated lateral translation of two centimeters to each side and up to four degrees of pelvic rotation (= FDhip); “pelvis and cuffs free”: both translations at the level of the cuffs and the pelvis were enabled (= FDboth). The settings were chosen in a way that patients should feel the most support/guidance during the *Guidance Force* condition and should feel the most freedom in condition FDboth.

During all conditions, we measured muscular activity of the following five muscles of the more affected leg by surface electromyography (sEMG): the M. rectus femoris (RF), M. vastus medialis (VM), M. biceps femoris, long head (BF), M. tibialis anterior (TA), and M. gastrocnemius lateralis (GL). Additionally, heart rate was measured with a heart rate belt with a sampling frequency of 0.2 Hz. Further details about the measurement equipment and methods can be found in the previous paper (Aurich-Schuler et al., 2017). All source data can be found in the Supplementary Material.

### Data Analysis and Statistics

#### Data Processing and Analysis

For the sEMG data analysis, we analyzed 10 strides (Shiavi et al., 1998) after 30 s of the start of each condition. Stance and swing phases were merged and data were rectified and smoothed by a Root Mean Square filter with a time window of 100 ms. For the heart rate, the values over the whole 2 min were averaged for each condition per participant and then averaged across participants.

For the quantitative sEMG data analysis, the amplitudes per step were averaged for each muscle and condition per participant (1 value per muscle per condition for each participant). These values were then averaged over all participants.

For the sEMG pattern analysis, the data from every step were merged and time-normalized to a linear envelope (1,000 samples, in which the normal stance-to-swing ratio of 60/40 was preserved, 100% gait cycle from heel strike to the next heel strike). To minimize between-subject variability, the envelopes were then amplitude-normalized to their maximal value (max. value = 100%) and an average over 10 envelopes/steps was taken per muscle and condition for each participant. From these averages, we took a grand-average over all participants. In our previous paper (Aurich-Schuler et al., 2017), a graphic illustration of these grand-averages of linear envelopes can be found in Figure 6 for the main conditions. For the interpretation of the physiological “normality” of the sEMG pattern, these grand-averages of each muscle per condition were compared to a reference curve of healthy children (Chang et al., 2007) with the Spearman correlation coefficient. This approach was chosen since participants were not able to elicit a “normal” gait pattern overground due to their disability. These Spearman correlations are also visible in Figure 6 of the previous paper (Aurich-Schuler et al., 2017).

For this article, we performed two new data analyses. First, we now plotted the Spearman correlation coefficients and the average of these coefficients for each condition to facilitate a comparison across control strategies. The correlations of the sEMG comparisons were interpreted as follows (adopted from Evans, 1996): *r* < 0.20, “very weak”; 0.20–0.39, “weak”; 0.40–0.59, “moderate”; 0.60–0.79, “strong”; and 0.80–1.00 “very strong relationship.”

Second, to estimate the task complexity of a walking condition in robot-assisted gait training, we calculated the correlations of the sEMG linear envelopes over these 10 consecutive steps for each condition (which we called inter-step similarity). We did this by correlating the linear envelope of every single step with that of all other steps using Spearman correlations and averaging this to one value per muscle and condition. This was done for the stance and swing phase separately. A high correlation value accordingly indicates a high similarity of sequential muscle activity patterns. We hypothesized that this value would be high in easy conditions and lower in more complex (variable) conditions. The hypothesis stems from the clinical observation that the imposed gait trajectory of the Lokomat is easier to follow if one offers the patient more support. By offering the patients more freedom (meaning less support/guidance by the robot), the adopted strategy to cope with this freedom depends on the gait capabilities of the patient. With high capabilities, it is easier to maintain a stable gait pattern even with more freedom, but with lower capabilities, patients will start coactivating other muscles to gain the necessary stability to maintain a constant gait pattern. Overall, the provision of more freedom is therefore hypothesized to lead to a lower interstep similarity which can therefore be seen as a reciprocal for variability.

#### Statistics

Statistics were done with IBM SPSS Statistics 25 (IBM Corporation, Armonk, NY, USA). Data were checked for normal distribution with the Shapiro-Wilk test together with Q-Q-plots and histograms. Because most of the data were not normally distributed, subsequent quantitative and pattern analyses were done with non-parametric tests. We applied Spearman correlations to examine the similarity of the sEMG patterns to the norm curves. Friedman tests were used to investigate the effect of the Lokomat conditions (independent variable) on the sEMG amplitudes (dependent variable) for each muscle and for heart rate independently and, in case of significant effects, Wilcoxon-tests were done for the pairwise *post-hoc* comparisons. Friedman tests were also used to investigate if the values of interstep-similarity (dependent variable) changed or remained constant across the seven conditions (independent variable). The significance level was set at α = 5%. Post-hoc corrections for multiple testing were done by calculating False Discovery Rate-corrected *p*-values (FDR) (Benjamini and Hochberg, 1995). Additionally, effect sizes were calculated (*r* = Z/√n) and scored according to Cohen's *d*: *d* = 0.1 is small, *d* = 0.3 is medium, and *d* = 0.5 is considered a large effect size (Cohen, 1988).

## Results

### Quantitative Analysis of the sEMG Data

#### Comparison Over All Conditions With Increasing Kinematic Variability

When comparing the averages of the sEMG amplitudes of the seven conditions with increasing kinematic freedom, the Friedman tests yielded significant results for a majority of muscles as well as for heart rate (see Table 1). Thereby, *Guidance Force* elicited the lowest amplitude in almost all muscles, as has been shown earlier (Aurich-Schuler et al., 2017).

**Table 1:** sEMG amplitudes of all conditions.

	**Condition**	**Median of amplitude [μV]**	**Interquartile range**	**Friedman test**
*M. rectus* femoris	GF	12.6	21.6	χ^2^ = 27.1 *p* < 0.001
	PC100	24.3	15.4	
	PC50	27.3	13.4	
	PC0	26.7	11.1	
	FreeDknee	21.8	19.6	
	FreeDhip	21.8	21.5	
	FreeDboth	22.5	14.3	
*M. vastus* medialis	GF	28.2	50.3	χ^2^ = 23.5 *p* = 0.001
	PC100	31.3	33.4	
	PC50	33.7	39.7	
	PC0	41.8	35.4	
	FreeDknee	36.9	66.8	
	FreeDhip	40.3	42.7	
	FreeDboth	38.8	41.8	
*M. biceps* femoris	GF	12.6	10.0	χ^2^ = 12.6 *p* = 0.05
	PC100	14.8	10.0	
	PC50	13.0	9.7	
	PC0	14.0	12.6	
	FreeDknee	14.1	11.9	
	FreeDhip	12.0	12.0	
	FreeDboth	15.6	10.0	
*M. tibialis* anterior	GF	23.2	36.1	χ^2^ = 19.3 *p* = 0.004
	PC100	29.8	41.3	
	PC50	41.3	48.2	
	PC0	39.5	45.9	
	FreeDknee	30.6	35.7	
	FreeDhip	35.5	40.8	
	FreeDboth	33.8	34.4	
*M. gastrocnemius* lateralis	GF	21.8	22.9	χ^2^ = 6.4 *p* = 0.39
	PC100	18.9	20.2	
	PC50	19.8	16.3	
	PC0	20.8	18.0	
	FreeDknee	18.8	19.5	
	FreeDhip	19.8	23.1	
	FreeDboth	21.6	23.7	
Heart rate	GF	100	25	χ^2^ = 32.5 *p* < 0.001
	PC100	106	25	
	PC50	108	22	
	PC0	109	25	
	FreeDknee	105	25	
	FreeDhip	106	25	
	FreeDboth	106	27	

*GF, Guidance Force; PC, Path Control; PC100 (and PC50 and PC0, respectively), Path Control with support force 100% (and 50 and 0%, respectively); FreeDknee, cuffs laterally moveable, pelvis fixated; FreeDhip, active pelvic translation/rotation, cuffs fixated; FreeDboth, cuffs laterally moveable and active pelvic translation/rotation*.

#### Path Control Sub-conditions

When focusing on sub-conditions of *Path Control*, the highest muscle activity was observable with a support force of 0%, which is the sub-condition with the maximum kinematic freedom in this condition. The results show that an increase in kinematic freedom led to a change in heart rate and muscle activity in all muscles except for the M. gastrocnemius lateralis (Friedman tests: M. rectus femoris: χ^2^ = 19.6, *p* < 0.001; M. vastus medialis: χ^2^ = 9.7, *p* = 0.008; M. biceps femoris: χ^2^ = 10.1, *p* = 0.006; M. tibialis anterior: χ^2^ = 8.8, *p* = 0.012; M. gastrocnemius lateralis: χ^2^ = 3.5, *p* = 0.17; Heart rate: χ^2^ = 17.7, *p* < 0.001). Table 2 shows pairwise post-hoc comparisons (Wilcoxon test) within the *Path Control* condition. Furthermore, significant results could be substantiated by medium to large effect sizes between the PC100 and PC0 conditions, which are the highest and lowest conditions concerning supportive force (Table 2).

**Table 2:** Comparison of sEMG amplitudes between different path control conditions (PC100, PC50, PC0).

	**Comparisons**	**FDR corrected *p*-values**	**Effect sizes (Cohen's *d*)**
*M. rectus* femoris	PC100-PC50	**0.013**	−0.48^*^
	PC50-PC0	0.061	−0.34^*^
	PC100-PC0	**0.003**	−0.61^**^
*M. vastus* medialis	PC100-PC50	0.112	−0.29
	PC50-PC0	**0.012**	−0.49^*^
	PC100-PC0	**0.009**	−0.54^**^
*M. biceps* femoris	PC100-PC50	0.211	−0.23
	PC50-PC0	0.091	−0.37^*^
	PC100-PC0	0.091	−0.34^*^
*M. tibialis* anterior	PC100-PC50	0.133	−0.29
	PC50-PC0	0.082	−0.38^*^
	PC100-PC0	**0.015**	−0.56^**^
*M. gastrocnemius* lateralis	PC100-PC50	0.308	−0.21
	PC50-PC0	0.176	−0.34^*^
	PC100-PC0	0.176	−0.31^*^
Heart rate	PC100-PC50	**0.007**	−0.52^**^
	PC50-PC0	0.147	−0.26
	PC100-PC0	**0.003**	−0.62^**^

P-values of the Wilcoxon test after FDR correction for multiple testing and effect sizes are shown. Statistically significant data are indicated in bold and medium effect sizes are marked with a ^*^, large effect sizes with ^**^. FDR, False Discovery Rate; PC100 (and PC50 and PC0, respectively), Path Control with support force 100% (and 50 and 0%, respectively).

#### FreeD Sub-conditions

In the sub-conditions of *FreeD*, no significant difference between the settings could be found (Friedman tests: M. rectus femoris: χ^2^ = 0.0, *p* = 1.0; M. vastus medialis: χ^2^ = 1.7, *p* = 0.42; M. biceps femoris: χ^2^ = 2.1, *p* = 0.34; M. tibialis anterior: χ^2^ = 3.2, *p* = 0.20; M. gastrocnemius lateralis: χ^2^ = 0.5, *p* = 0.78; Heart rate: χ^2^ = 0.9, *p* = 0.63).

### Pattern Analysis of the sEMG Data

**Figure 2:**
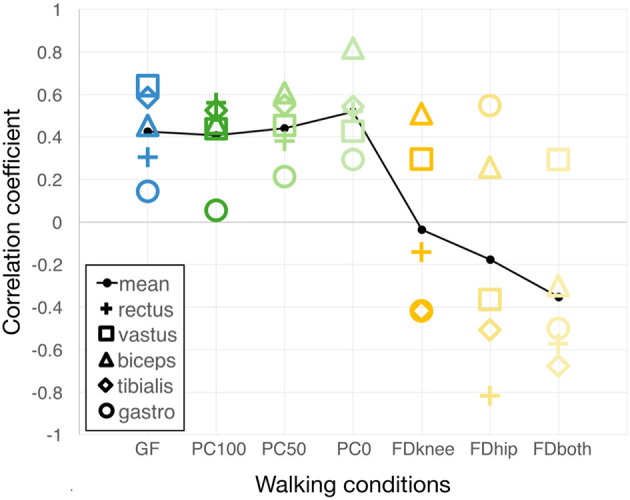
Correlations of grand-averaged gait cycle sEMG patterns with norm patterns. For each condition, the sEMG pattern of the grand-averaged gait cycle of each measured muscle was correlated with the respective norm pattern from Chang et al. (2007). Higher values accordingly denote a more similar muscle activity pattern compared to that of healthy children walking overground. The black line connects the average values of all correlations of the five measured muscles. GF, Guidance Force; PC, Path Control; FD, FreeD; PC100 (and PC50 and PC0, respectively), Path Control with support force 100% (and 50 and 0%, respectively); FDknee, cuffs laterally moveable, pelvis fixated; FDhip, active pelvic translation/rotation, cuffs fixated; FDboth, cuffs laterally moveable and active pelvic translation/rotation; rectus, M. rectus femoris; vastus, M. vastus medialis; biceps, M. biceps femoris; tibialis, M. tibialis anterior; gastro M. gastrocnemius lateralis.

An extensive visual representation of the sEMG gait cycle patterns is provided in the first paper (Aurich-Schuler et al., 2017). Figure 2 of the current manuscript visualizes the Spearman correlation coefficients of the grand-averaged gait cycle sEMG patterns with norm patterns of healthy children (the numeric values of the main conditions GF, PC100, and FDboth can also be found in Figure 6 of the first paper Aurich-Schuler et al., 2017). In *Path Control* sub-conditions, it is observable that a decrease in support force goes along with a higher correlation with the norm. In contrast, *FreeD* sub-conditions with higher kinematic freedom showed lower correlation coefficients.

**Figure 3:**
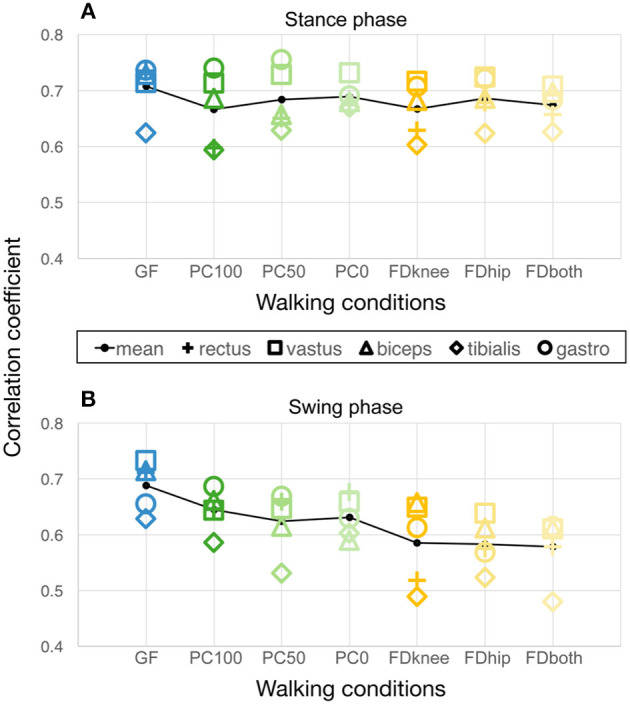
Inter-step similarity of muscle activity patterns over 10 steps during **(A)** stance phase and **(B)** swing phase, respectively. The black line connects the average values of all correlations of the five measured muscles. GF, Guidance Force; PC, Path Control; FD FreeD; PC100 (and PC50 and PC0, respectively), Path Control with support force 100% (and 50 and 0%, respectively); FDknee, cuffs laterally moveable, pelvis fixated; FDhip, active pelvic translation/rotation, cuffs fixated; FDboth, cuffs laterally moveable and active pelvic translation/rotation; rectus, M. rectus femoris; vastus, M. vastus medialis; biceps, M. biceps femoris; tibialis, M. tibialis anterior; gastro, M. gastrocnemius lateralis.

The inter-step similarity as a reciprocal of step variability is shown in Figure 3. Figure 3A shows that the inter-step similarity during stance phase, where the timing of muscle activity is influenced by the treadmill rather than the control strategy, remains constant across conditions (Friedman Test: χ^2^ = 5.5, *p* = 0.48) with moderate to strong correlations (averaged Spearman correlation coefficient during GF: ρ = 0.71 ± 0.05; PC100: ρ = 0.67 ± 0.07; PC50: ρ = 0.68 ± 0.05; PC0: ρ = 0.69 ± 0.02; FDknee: ρ = 0.67 ± 0.05; FDhip: ρ = 0.69 ± 0.04; FDboth: ρ = 0.67 ± 0.03). During the swing phase (Figure 3B), however, the inter-step similarity significantly decreases (Friedman Test: χ^2^ = 17.9, *p* = 0.006) as the kinematic freedom increases (GF: ρ = 0.69 ± 0.04; PC100: ρ = 0.64 ± 0.04; PC50: ρ = 0.62 ± 0.06; PC0: ρ = 0.63 ± 0.04; FDknee: ρ = 0.59 ± 0.08; FDhip: ρ = 0.58 ± 0.04; FDboth: ρ = 0.58 ± 0.06).

## Discussion

This secondary data report investigates alterations in sEMG activity levels and patterns induced by technical features with different degrees of support, and therefore, different levels of kinematic freedom. While the first article only contained data of one condition of each of the control strategies *Guidance Force, Path Control, and FreeD* (Aurich-Schuler et al., 2017), this secondary data analysis offers a much more detailed perspective on the differences between these strategies and introduces the new metric of inter-step similarity.

### Quantitative Analysis of the sEMG Data

In general, we could observe that the different sub-conditions elicited higher muscular efforts compared to the standard *Guidance Force* condition, except for M. gastrocnemius lateralis. It makes sense that the M. gastrocnemius lateralis remains unaffected by the variation of conditions in this study, as this muscle is predominantly active during the stance phase, whereas *Path Control* rather influences muscle activity during swing phase of the gait cycle and FreeD does not primarily act on the level of the foot.

While *Path Control* sub-conditions differed significantly (except for the M. gastrocnemius lateralis), variations in *FreeD* settings did not have a differential influence on the amplitude of the investigated muscles. However, it can be argued that alterations in *FreeD* settings would rather project to changes in trunk and pelvic muscles rather than the leg muscles measured in this project. Further studies are needed to investigate how the different *FreeD* settings impact muscular activation, but also kinematics in patients. Two-dimensional kinematic analysis of *FreeD* in healthy participants has been done and it was shown that the *FreeD* increases lateral translation of the pelvis and reduces compensatory movements of the upper body (Aurich-Schuler et al., 2019). However, healthy participants can obviously deal with the provided kinematic freedom at the pelvis, whereas patients might sometimes need more guidance to achieve a functional walking pattern. A differentiated analysis in patients could shed light on how to optimally use the *FreeD* settings and if it makes sense at all to allow translation of the thighs while having the pelvis fixated or vice versa. In any case, as outlined in the next paragraph, participants could better deal with variations of kinematic freedom on the sagittal plane (*Path Control*, at the level of the leg/foot) as on the frontal plane (*FreeD*, at the level of the pelvis/thigh).

### Pattern Analysis of the sEMG Data

As already emphasized in the first research paper (Aurich-Schuler et al., 2017), it is not always meaningful to strive for maximization of muscle activity, especially if it does not occur at the right time during the gait cycle. This particularly applies to children with cerebral palsy. Cerebral palsy is usually accompanied by muscular weakness, spasticity, and contractures, which result in a poor biomechanical alignment of the legs and increases the metabolic cost of walking (Perry, 1992). Hence, patients with cerebral palsy often walk with increased muscle activity and heart rate. Therefore, in the current work, different pattern analyses to interpret the physiological “normality” and variability of the activity patterns (per muscle and per control strategy) were performed.

Figure 2 shows that muscles react differently to the increase of kinematic freedom within *Path Control* and *FreeD*. An increase in kinematic freedom led to higher correlation coefficients in *Path Control* conditions. They were even higher compared to those elicited by *Guidance Force* (still the clinically mostly applied control mechanism) which has shown to elicit reasonably similar patterns compared to overground walking in an earlier study (Aurich Schuler et al., 2013), as confirmed by this study. This supports the assumption that *Guidance Force* might train gait skills rather in a restorative than compensatory way (Aurich-Schuler et al., 2017), and it appears that *Path Control* shows similar tendencies.

In contrast, patterns of many muscles during *FreeD* showed poor correlations with the norm walking pattern, irrespective of the settings of the sub-conditions. Furthermore, *FreeD* elicited an increased inter-muscular variability in the correlations with the norm curves (Figure 2), but also an increased intra-muscular variability between individual steps (Figure 3). A clear difference to *Path Control* is apparent, and there is mainly one reason for this. The *FreeD* increases the possible degrees of freedom by enabling movement on the frontal plane at the level of the pelvis and thigh. This is potentially destabilizing and requires weight shifting as the lateral support of the Lokomat at the knee and hip is no longer provided. *Path Control*, however, introduces a possible deviation from the predefined foot trajectory only in the sagittal plane, which, for a patient, may not be so difficult to manage. Accordingly, walking with *FreeD* is probably just more difficult/too complex as more degrees of freedom need to be controlled with potentially impaired muscles. This is nicely reflected in Figure 3, which shows a decreased inter-step similarity in FreeD conditions, as initially hypothesized.

It must be kept in mind that the Lokomat with all its boundaries tries to facilitate “normal” walking and that most of the patients included in this study are not able to produce “normal” activity patterns when walking overground. Accordingly, the increase in variability after the removal of the lateral stabilization/fixation could also reflect a return to the patients' habitual (compensatory) activity patterns, rather than a loss of control. Consequently, it might be, that *FreeD* enables a more habitual and not restorative gait pattern due to the allowed degrees of freedom. It, therefore, could make sense to use *FreeD* in populations, who already have quite a physiological habitual gait pattern, and to use *Path Control* in populations, where the restoration of a “normal” gait pattern is important and possible, for instance in patients with acquired brain injury. Further studies are necessary to look into this possibly very relevant aspect of the different control strategies and technical features.

From a motor learning perspective, the *FreeD* goes in the right direction. Wu et al. assumed in their work that robotic therapies with fixed trajectory control strategies might not be optimally effective to increase walking functions in children with cerebral palsy (Wu et al., 2017). The reason might lie in the absence of training of lateral weight shift and variability in limb kinematics. They concluded that variability in leg and pelvis kinematics might facilitate the transfer of motor skills from treadmill therapies to overground walking (Wu et al., 2017). This is in accordance with Aoyagi et al. (2007) who mentioned that it is important for a natural gait that the robot device allows the leg to abduct and the pelvis to rotate and make a lateral translation. Also, Koopman et al. (2013) suggested in their experimental work that lateral balance control requires more active muscular participation than sagittal balance. However, the *FreeD* study in healthy participants showed no difference in trunk muscle activation compared to walking without the *FreeD* module (Aurich-Schuler et al., 2019). Further studies are needed to investigate if the driven actuation of the *FreeD* module actually even hinders active weight shifting and enables slacking in that dimension (Reinkensmeyer et al., 2009).

These uncertainties stress once again the importance of the therapist's knowledge and perception to implement this tool optimally according to the patient's capabilities (Aurich-Schuler et al., 2017). He/she should know the technical possibilities and limitations and has to interpret how a patient reacts to different modalities (is a patient still challenged or already overstrained?). However, in the future, the therapist could be supported with the new metric of inter-step similarity to determine the optimal control strategy for each patient, and therefore tailoring the therapy to the individual possibilities of the patient. In our study, this was not the case, since we predefined several sub-conditions and, therefore, limited the therapist's options to respond individually to the reaction and performance of the patient, and adjust modulations or provide more verbal support. Accordingly, we hypothesize that the chosen extreme settings for the *FreeD* condition in this study contributed to the high variability of the sEMG patterns by applying a fix set of parameters to patients that were not always able to meet the given demands. Further studies should investigate the usefulness of the inter-step similarity metric as an indicator for adequate support of the Lokomat.

### Clinical Implications

Results from this study may have some clinical implications. Practical application of the sub-conditions has shown that the basic settings may have been too extreme. As mentioned above and discussed previously (Aurich-Schuler et al., 2017), 100% support force during PC100 combined with a big tunnel was probably confusing. This sub-condition allows a high spatial variability together with a minimum of temporal variability. With our experience in therapy and research, we propose to select a support force between 20 and 80%. Additionally, a Guidance Force of 0% was too little, as the Lokomat then only compensates for gravity and Coriolis forces, but not for inertia. Consequently, Krishnan et al. (2013) suggested training with at least 10% Guidance Force parallel to *Path Control* to overcome the inertia of the Lokomat. Even though we cannot confirm that 10% is the right choice, we can partially support the recommendations (Aurich-Schuler et al., 2017). With our current experience, we suggest applying ~35% Guidance Force for children and adolescents with neurological gait disorders.

Further, a drawback of the *FreeD* in its current form is that the pelvic *FreeD* module is actuated. This means that translation and rotation of the pelvis are being guided by the device and the patients do not have to actively perform these movements. Accordingly, one has to be aware that this can have an influence on the amplitude and the pattern of muscle activity.

## Limitations

The analyses of this report base on the data acquisition of our main research project, where several limitations have already been discussed (Aurich-Schuler et al., 2017). We like to stress that the patient population surveyed is very heterogeneous but reflects the patient pool that we experience in our everyday clinical routine. Nevertheless, the subpopulation of patients who participate in Lokomat therapies is again relatively homogeneous due to the inclusion criteria of the robotic device. Furthermore, general statements about the technical features of such devices are relevant and justified in our opinion, as it helps to understand the technology and its application in therapy.

We hypothesized that outcome measure of interstep similarity was reflective of task complexity. While the results seem to confirm this, it might also be that other factors have led to the decrease in interstep similarity with higher kinematic freedom. Further studies need to investigate the validity of this approach.

The settings chosen in this study had the aim to allow the technologies to fully unfold their potential to facilitate their exploration. Please be aware that these settings, in most cases, are not recommended for regular clinical therapy. Additionally, durations of conditions were rather short and we might therefore have missed even larger adaptations of muscle activity. However, and this is the most important part, the transfer of this knowledge into therapy has to be finally done individually for each patient. This requires very experienced therapists who understand the technology they are working with.

Finally, the first versions of the *FreeD* module allowed a translational movement of the cuffs only in the medial direction whereas a lateral movement of the leg during weight-shifting was mechanically prevented. This could have influenced our results. In the current version of the *FreeD* module, this issue has been solved.

## Conclusion

This secondary data analysis report focuses on six sub-conditions of *Path Control* and *FreeD*. In general, this work highlights that the new hard- and software approaches influence muscle activity differently. An increase of kinematic freedom of the walking condition either led to an increase in muscular effort (*Path Control*) or to a higher step variability (*FreeD*). The latter can be interpreted as an increased task complexity of this condition. We would like to reiterate that the therapist plays a crucial role in robot-assisted therapy. It is the therapist's judgment to decide which technical approach is to be the best for the individual patient's capabilities or the time frame during a patient's rehabilitation progress.

## Data Availability Statement

The datasets supporting the conclusions of this article can be found in the supplementary material of this article. Complementing datasets can be found in the supplementary material of the first research article (Aurich-Schuler et al., 2017).

## Ethics Statement

The studies involving human participants were reviewed and approved by Cantonal Ethics Committee of Zurich, Switzerland. Written informed consent to participate in this study was provided by the participants' legal guardian/next of kin.

## Author Contributions

TA-S: research project conception and execution, data acquisition review and critique, and manuscript writing. RL: research project conception, statistical analysis, manuscript writing, review, and critique. Both authors approved the final manuscript.

### Conflict of Interest

The authors declare that the research was conducted in the absence of any commercial or financial relationships that could be construed as a potential conflict of interest.

## Supplementary Material

Supplementary Data Sheet 1 STROBE checklist.

Supplementary Data Sheet 2 Source data sEMG Path Control 50%.

Supplementary Data Sheet 3 Source data sEMG Path Control 0%.

Supplementary Data Sheet 4 Source data sEMG FreeD knee.

Supplementary Data Sheet 5 Source data sEMG FreeD hip.

Supplementary Data Sheet 6 Source data Heart rate.

## References

[B1] Aoyagi D. Ichinose W. E. Harkema S. J. Reinkensmeyer D. J. Bobrow J. E. (2007). A robot and control algorithm that can synchronously assist in naturalistic motion during body-weight-supported gait training following neurologic injury. IEEE Trans. Neural Syst. Rehabil. Eng. 15, 387–400. 10.1109/TNSRE.2007.903922 17894271

[B2] Aurich Schuler T. Müller R. van Hedel H. J. (2013). Leg surface electromyography patterns in children with neuro-orthopedic disorders walking on a treadmill unassisted and assisted by a robot with and without encouragement. J. Neuroeng. Rehabil. 10:78. 10.1186/1743-0003-10-78 23867005 PMC3720176

[B3] Aurich-Schuler T. Grob F. van Hedel H. J. A. Labruyère R. (2017). Can Lokomat therapy with children and adolescents be improved? An adaptive clinical pilot trial comparing guidance force, path control, and FreeD. J. Neuroeng. Rehabil. 14:76. 10.1186/s12984-017-0287-1 28705170 PMC5513325

[B4] Aurich-Schuler T. Gut A. Labruyère R. (2019). The FreeD module for the Lokomat facilitates a physiological movement pattern in healthy people – a proof of concept study. J. Neuroeng. Rehabil. 16:26 10.1186/s12984-019-0496-x 30728040 PMC6366098

[B5] Aurich-Schuler T. Warken B. Graser J. V. Ulrich T. Borggraefe I. Heinen F.. (2015). Practical recommendations for robot-assisted treadmill therapy (Lokomat) in children with cerebral palsy: indications, goal setting, and clinical implementation within the WHO-ICF framework. Neuropediatrics 46, 248–260. 10.1055/s-0035-1550150 26011438

[B6] Benjamini Y. Hochberg Y. (1995). Controlling the false discovery rate: a practical and powerful approach to multiple testing. J. R. Stat. Soc. Ser. B 57, 289–300. 10.1111/j.2517-6161.1995.tb02031.x

[B7] Chang W. N. Lipton J. S. Tsirikos A. I. Miller F. (2007). Kinesiological surface electromyography in normal children: range of normal activity and pattern analysis. J. Electromyogr. Kinesiol. 17, 437–445. 10.1016/j.jelekin.2006.02.003 16603385

[B8] Cohen J. (1988). Statistical Power Analysis for the Behavioral Sciences, 2nd Edn Hillsdale, NJ: Lawrence Erlbaum Associates, Inc.

[B9] Dundar U. Toktas H. Solak O. Ulasli A. M. Eroglu S. (2014). A comparative study of conventional physiotherapy versus robotic training combined with physiotherapy in patients with stroke. Top. Stroke Rehabil. 21, 453–461. 10.1310/tsr2106-453 25467393

[B10] Duschau-Wicke A. Caprez A. Riener R. (2010a). Patient-cooperative control increases active participation of individuals with SCI during robot-aided gait training. J. Neuroeng. Rehabil. 7:43. 10.1186/1743-0003-7-43 20828422 PMC2949707

[B11] Duschau-Wicke A. von Zitzewitz J. Caprez A. Lunenburger L. Riener R. (2010b). Path control: a method for patient-cooperative robot-aided gait rehabilitation. IEEE Trans. Neural Syst. Rehabil. Eng. 18, 38–48. 10.1109/TNSRE.2009.2033061 20194054

[B12] Evans J. D. (1996). Straightforward Statistics for the Behavioral Sciences. Pacific Grove, CA: Brooks/Cole Pub. Co; An International Thomsom Publ. Co.

[B13] Koopman B. Meuleman J. H. van Asseldonk E. H. F. van der Kooij H. (2013). Lateral balance control for robotic gait training, in 2013 IEEE 13th International Conference on Rehabilitation Robotics (ICORR) (Seattle, WA: IEEE), 1–6. 10.1109/ICORR.2013.6650363 24187182

[B14] Krishnan C. Ranganathan R. Dhaher Y. Y. Rymer W. Z. (2013). A pilot study on the feasibility of robot-aided leg motor training to facilitate active participation. PLoS ONE 8:e77370. 10.1371/journal.pone.0077370 24146986 PMC3795642

[B15] Mehrholz J. Thomas S. Werner C. Kugler J. Pohl M. Elsner B. (2017). Electromechanical-assisted training for walking after stroke. Cochrane Database Syst. Rev. 5:CD006185. 10.1002/14651858.CD006185.pub4 28488268 PMC6481755

[B16] Palisano R. Rosenbaum P. Walter S. Russell D. Wood E. Galuppi B. (1997). Development and reliability of a system to classify gross motor function in children with cerebral palsy. Dev. Med. Child Neurol. 39, 214–223. 10.1111/j.1469-8749.1997.tb07414.x 9183258

[B17] Perry J. (1992). Gait Analysis: Normal and Pathological Function. Thorofare, NJ: SLACK Incorporated.

[B18] Reinkensmeyer D. J. Akoner O. M. Ferris D. P. Gordon K. E. (2009). Slacking by the human motor system: computational models and implications for robotic orthoses, in 2009 Annual International Conference of the IEEE Engineering in Medicine and Biology Society (Minneapolis, MN: IEEE), 2129–2132. 10.1109/IEMBS.2009.5333978 19964581

[B19] Riener R. Lünenburger L. Jezernik S. Anderschitz M. Colombo G. Dietz V. (2005). Patient-cooperative strategies for robot-aided treadmill training: first experimental results. IEEE Trans. Neural Syst. Rehabil. Eng. 13, 380–394. 10.1109/TNSRE.2005.848628 16200761

[B20] Schück A. Labruyère R. Vallery H. Riener R. Duschau-Wicke A. (2012). Feasibility and effects of patient-cooperative robot-aided gait training applied in a 4-week pilot trial. J. Neuroeng. Rehabil. 9:31. 10.1186/1743-0003-9-31 22650320 PMC3533836

[B21] Shiavi R. Frigo C. Pedotti A. (1998). Electromyographic signals during gait: criteria for envelope filtering and number of strides. Med. Biol. Eng. Comp. 36, 171–178. 10.1007/BF02510739 9684456

[B22] van Hedel H. J. A. Aurich-Schuler T. (2016). Clinical application of rehabilitation technologies in children undergoing neurorehabilitation, in Rehabilitation Technology, edsReinkensmeyer D. J. Dietz V. (Cham: Springer International Publishing), 283–308. 10.1007/978-3-319-28603-7_14

[B23] Wu M. Kim J. Arora P. Gaebler-Spira D. J. Zhang Y. (2017). Effects of the integration of dynamic weight shifting training into treadmill training on walking function of children with cerebral palsy. Am. J. Phys. Med. Rehabil. 96, 765–772. 10.1097/PHM.0000000000000776 28644244 PMC5648612

